# Prevalence and clinical characteristics of blown-out myotomy after E-POEM for achalasia: a systematic review and meta-analysis

**DOI:** 10.1007/s00464-026-12950-y

**Published:** 2026-06-03

**Authors:** Yusuf Kagzi, Srinivas Reddy Puli

**Affiliations:** University of Illinois College of Medicine Peoria, Peoria, IL USA

**Keywords:** Achalasia, Peroral endoscopic myotomy, Blown-out myotomy, Esophageal motility disorder, Eckardt score, Meta-analysis

## Abstract

**Background:**

Achalasia is a primary esophageal motility disorder characterized by impaired lower esophageal sphincter relaxation and absent peristalsis. Treatment options include Pneumatic Dilation, Laparoscopic Heller Myotomy, and Esophageal Peroral Endoscopic Myotomy (E-POEM). A focal distal esophageal dilation at the myotomy site, termed blown-out myotomy (BOM), has been reported in 9% – 32% of patients and may be associated with reflux, esophageal retention, and treatment failure. The clinical significance of BOM remains unclear. This meta-analysis evaluated the prevalence and clinical characteristics of BOM following E-POEM.

**Methods:**

A systematic search of electronic databases and conference proceedings from inception through November 2025 identified prospective and retrospective studies reporting the prevalence or clinical features of BOM after E-POEM. The primary outcome was pooled BOM prevalence. Secondary outcomes included symptom severity (Eckardt score), myotomy characteristics, and associations with achalasia subtypes. Studies reporting ≤ 3 BOM cases or lacking extractable data were excluded. Pooled proportions were calculated using a fixed-effects model. Publication bias was assessed using the Begg–Mazumdar test. Mean Eckardt scores were compared using an unpaired t-test.

**Results:**

Four studies comprising 947 patients were included. The pooled prevalence of BOM was 11.16% (95% CI: 9.28% –13.25%). No evidence of publication bias was detected (Kendall’s tau-*b* = 0.33; *p* = 0.75). Baseline characteristics were comparable between groups. Mean myotomy length was 10.39 ± 1.82 cm in the BOM group and 11.23 ± 1.46 cm in the non-BOM group. Pretreatment Eckardt scores were similar (6.67 vs 6.63), as were 3-month post–E-POEM scores (2.05 vs 1.93). Pooled odds ratios for BOM were 0.73 (95% CI: 0.47–1.13) for type I achalasia, 0.79 (95% CI: 0.53–1.19) for type II, and 1.38 (95% CI: 0.80–2.38) for type III.

**Conclusions:**

BOM occurs in a minority of patients following E-POEM. Across studies, BOM did not show consistent associations with symptoms or procedural characteristics.

Achalasia is a primary esophageal motility disorder characterized by impaired lower esophageal sphincter (LES) relaxation and absent peristalsis, leading to dysphagia, regurgitation, chest pain, and weight loss. The disease is thought to result from autoimmune-mediated loss or dysfunction of enteric neurons within the esophageal myenteric plexus [1]. Although no therapy reverses the underlying pathophysiology, current treatments including Pneumatic Dilation, Laparoscopic Heller myotomy (LHM), and Esophageal Peroral Endoscopic Myotomy (E-POEM) aims to mechanically disrupt the LES to reduce outflow obstruction and improve esophageal emptying. These approaches achieve long-term clinical success rates of approximately 80%–90% following myotomy-based therapy [2–4].

Nevertheless, symptom recurrence or treatment failure after myotomy occurs in 10%-20% of patients after myotomy and has traditionally been attributed to incomplete division of the LES or persistent esophagogastric junction (EGJ) outflow obstruction. However, prior studies reported that some patients continue to experience symptoms despite a technically complete myotomy confirmed by high-resolution manometry and functional lumen imaging probe evaluation (FLIP). This observation highlights the importance of esophageal body function in effective bolus transit. Structural and functional abnormalities of the esophageal body including impaired or disordered contractility, esophageal dilatation, peptic strictures, diverticulum formation, and severe tortuosity may contribute to persistent esophageal retention and symptom recurrence even after successful completion of a myotomy [5–7].

An increasingly reported anatomic finding after E-POEM is focal distal esophageal dilation at the myotomy site, commonly referred to as blown-out myotomy (BOM) or pseudodiverticulum. This phenomenon was first described in 1988 by Rubesin et al. as “distal esophageal ballooning” following LHM. BOM is characterized by a wide-mouthed, diverticular-like outpouching in the distal esophagus and may predispose to food stasis and recurrent symptoms, potentially reflecting altered esophageal emptying dynamics despite an otherwise adequate myotomy. Endoscopically, BOM is described across four grades of severity: Grade 0, no diverticular-like changes; Grade 1, mild focal dilation; Grade 2, diverticular-like changes with septum formation; and Grade 3, food retention. Grades 2 and 3 are defined as endoscopic BOM. Radiographically, BOM is defined on timed barium esophagram (TBE) as a greater than 50% increase in distal esophageal diameter within 5 cm proximal to the lower esophageal sphincter. [7–12].

Reported prevalence of BOM varies widely across cohorts, ranging from 9% to over 30% following E-POEM or LHM. Prior studies have reported mixed findings, with BOM described as either a benign postoperative change or associated with increased symptoms and treatment failure. Proposed contributors to BOM development include elevated post-treatment integrated relaxation pressure (IRP), type III achalasia physiology, and longer or anterior myotomies, although these associations have not been consistently demonstrated. Moreover, data specifically focusing on the occurrence and progression of BOM following E-POEM alone remain limited [10, 13, 14].

Given the increasing recognition of BOM and the heterogeneity in reported prevalence and symptom associations, a clearer understanding of its frequency, clinical course, and relationship to patient- and procedure-level factors after E-POEM is needed. This meta-analysis aimed to estimate the prevalence of BOM in patients with achalasia and to summarize its associations with symptom severity, procedural characteristics, and achalasia subtype.

## Methods

### Search methodology

A comprehensive literature search was performed across multiple electronic databases, including PubMed, Embase, Cochrane Library (Central and Database of Systematic Reviews), Google Scholar, Scopus, and Web of Science from database inception through November 2025. The search strategy was developed in accordance with PRISMA (Preferred Reporting Items for Systematic Reviews and Meta-Analyses) guidelines and included relevant conference abstracts where available. Search terms and Medical Subject Headings (MeSH) included combinations of: *“achalasia,” “peroral endoscopic myotomy,” “POEM,” “esophageal myotomy,” “blown-out myotomy,” “pseudo-diverticulum,” “distal esophageal dilation,”* and *“epiphrenic diverticulum.”* Reference lists of retrieved articles were manually screened for additional eligible studies. Duplicate and overlapping data were excluded after thorough cross-checking.

### Study eligibility

Studies were eligible for inclusion if they reported outcomes related to BOM following E-POEM in adult human subjects. Eligible studies were required to report at least one relevant outcome. Articles were excluded if they were not published in English. Animal studies, case reports, editorials, review articles, and commentaries were excluded. Two authors (YK and SP) reviewed the full-text articles independently.

### Data extraction and quality assessment

Two authors (YK and SP) independently extracted data from included studies using a standardized data collection form. Extracted variables included: (a) study characteristics (first author, year of publication, study design, and country); (b) patient demographics (sample size, age, sex, and body mass index when available); (c) procedural characteristics (type of intervention, myotomy approach and length when reported, and follow-up duration); and (d) outcomes related to BOM, including prevalence or incidence, symptom severity as measured by the Eckardt score, IRP, achalasia subtype, and other reported clinical or radiographic findings. Any discrepancies in data extraction were resolved by consensus.

### Outcomes evaluated

The primary outcome was the prevalence of BOM following E-POEM. Secondary outcomes included symptom severity assessed by the Eckardt score, manometric parameters including IRP, procedural characteristics, and associations with achalasia subtype.

### Statistical analysis

This meta-analysis was performed by calculating weighted pooled effects. Individual study proportions were transformed into a quantity using the Freeman-Tukey variant of the arcsine square-root transformed proportion. The pooled proportion is calculated as the back-transform of the weighted mean of the transformed proportions, using inverse arcsine variance weights for the fixed-effects model and the random-effects model [15, 16]. The heterogeneity of the studies was evaluated using Cochran’s Q test, based on inverse variance weights, and by calculating the I^2^ statistic [17]. I^2^ values of 0–39% were considered as non-significant heterogeneity; 30–60% suggest moderate heterogeneity, 60–75% substantial heterogeneity, and > 75% considerable heterogeneity. A *p*-value > 0.10 rejects the null hypothesis that the studies are heterogeneous. The findings of this meta-analysis are reported using the fixed-effects model, as there was no statistically significant heterogeneity. Forest plots were drawn to show the point estimates in each study in relation to the summary of the pooled estimate. The width of point estimates in the Forest plots indicates the weight assigned to that study. The Egger bias indicator and Begg-Mazumdar bias indicator tested the effects of publication and selection bias on summary estimates [18, 19]. Funnel plots were constructed to assess potential publication bias [20, 21]. For continuous outcomes, unpaired t-tests were used to compare mean values between patients with BOM and those without BOM when data were available. All statistical analyses were performed using Microsoft Excel (Version 19).

## Results

### Study selection and characteristics

**Fig. 1:**
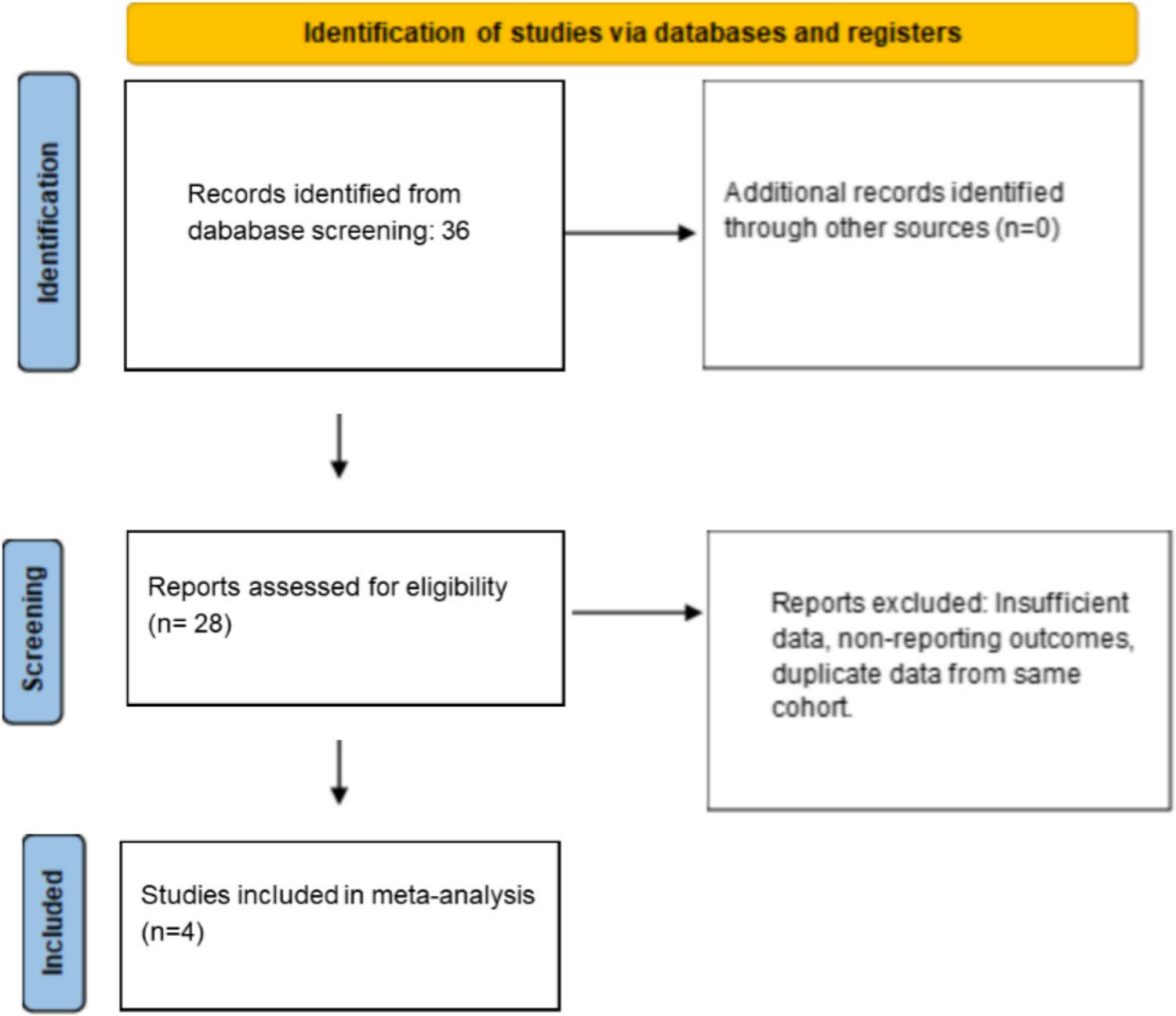
PRISMA flow diagram for the meta-analysis evaluating BOM following E-POEM

The initial literature search identified 36 studies, of which 12 full-text articles were reviewed in detail. A total of 4 studies met the inclusion criteria [10, 13, 22, 23]. The search strategy was developed with the assistance of a medical librarian. The study selection process is summarized in the PRISMA flow diagram (Fig. 1). The quality of studies was good, as evaluated using the modified Newcastle–Ottawa scale. All the pooled estimates given are estimates calculated using the fixed-effects model. The agreement between reviewers was 1.0, as measured by Cohen’s κ.

### Baseline characteristics

A total of 947 patients were included (BOM: 124/947). Across studies, the mean age was 46.24 ± 2.0 years in the BOM group and 51.36 ± 2.44 years in the non-BOM group. The mean body mass index was 22.55 ± 1.54 kg/m^2^ in the BOM group and 21.92 ± 2.63 kg/m^2^ in the non-BOM group. The mean follow-up duration across studies was approximately 39 months, with individual study follow-up ranging from 24 to 60 months.

### Prevalence of blown-out myotomy

**Fig. 2:**
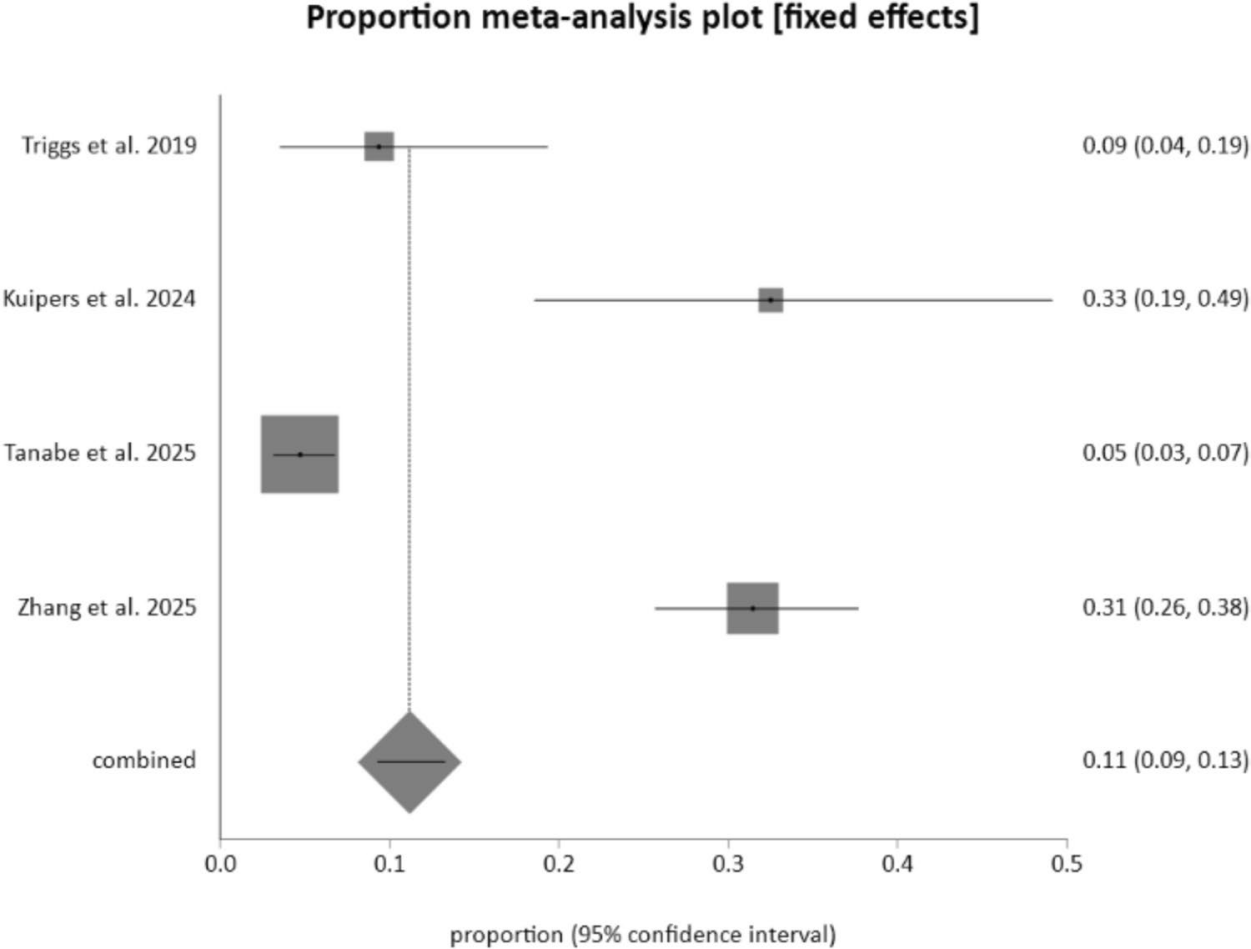
Forest plot showing individual study proportions and the weighted pooled prevalence of BOM following E-POEM

The pooled prevalence of BOM was 11.16% (95% CI: 9.28–13.25). There was no significant heterogeneity with an I^2^ = 0%. A forest plot displaying individual study estimates and the pooled estimate is shown in Fig. 2. The Begg-Mazumdar bias indicator yielded a Kendall’s tau *b* value of 0.33 (*p* = 0.75), indicating no publication bias. The pooled prevalence of BOM at 1 year was 17.5% (95% CI: 11.5%-24.5%). The pooled proportion without BOM was 88.8% (95% CI: 86.7%-90.8%).

### Procedural characteristics

Mean myotomy length was 10.39 ± 1.83 cm in the BOM group and 11.24 ± 1.46 cm in the non-BOM group. The mean IRP in patients with BOM was 27.0 mmHg preoperatively and 16.4 mmHg post-E-POEM.

### Symptom severity (Eckardt score)

Symptom severity was assessed by the Eckardt score. Mean pretreatment Eckardt scores were 6.67 in the BOM group and 6.63 in the non-BOM group. An unpaired Student’s t-test showed a mean difference of 0.03 (95% CI: − 2.57 to −2.64), with no evidence of variance inequality (two-sided F test not significant). At 3 months following E-POEM, Eckardt score showed mean scores of 2.05 in the BOM group and 1.93 in the non-BOM group. The between-group mean difference was 0.13 (95% CI: −2.71 to −2.96).

### Achalasia subtype and association with BOM

**Fig. 3:**
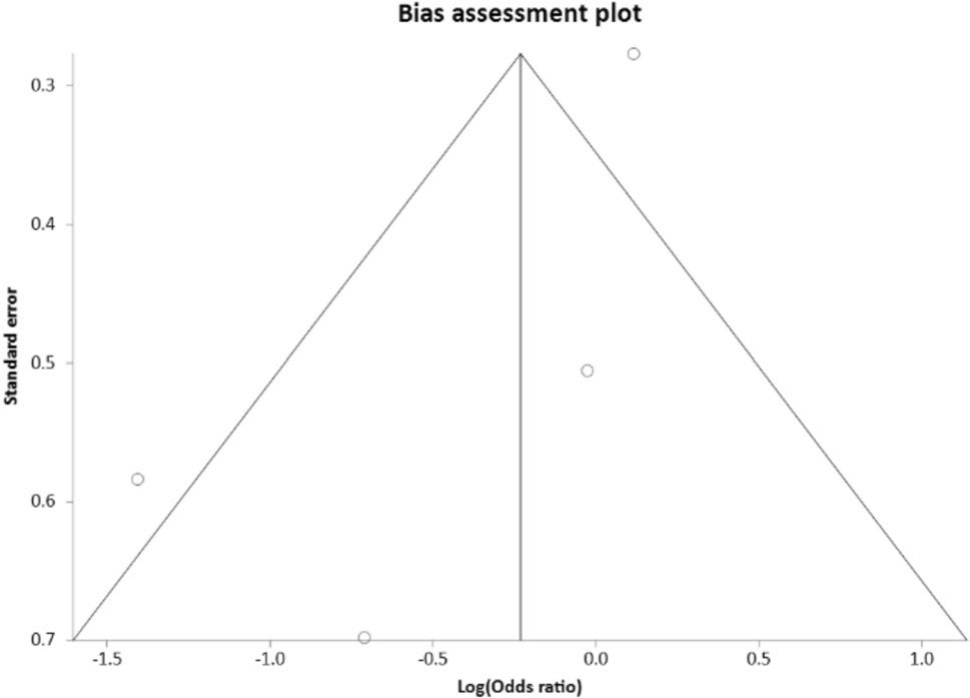
Funnel plot showing publication bias

Achalasia subtype distribution was evaluated in relation to BOM status. The pooled odds ratio for type I achalasia in patients with BOM compared to non–BOM group was 0.73 (95% CI: 0.47–1.13). For type II achalasia, the pooled odds ratio was 0.79 (95% CI: 0.53–1.19). For type III achalasia, the pooled odds ratio was 1.38 (95% CI: 0.80–2.38). Corresponding funnel plot assessing publication bias is shown in Fig. 3.

## Discussion

In this meta-analysis, we assessed the prevalence and clinical characteristics of BOM following E-POEM for achalasia. Our analysis showed that BOM occurred in approximately 11% of patients after E-POEM. Across individual studies, BOM prevalence ranges from 9% – 30%, reflecting differences in study design, patient populations, and follow-up duration. In the present analysis, the mean follow-up was about 39 months. Higher prevalence rates were observed in cohorts with longer follow-up durations, whereas lower in larger cohorts as seen in the studies by Tanabe et al. and Zhang et al. respectively [22, 23]. In the study by Tanabe et al., BOM was not detected at 3 months after E-POEM but was observed during follow-up between 1 and 3 years post-procedure [22]. Consistent with this time course, in our study BOM prevalence at 1 year was approximately 18%, supporting that BOM may develop or become radiographically apparent during longitudinal follow-up rather than immediately after intervention. This trend is further illustrated by data showing increasing BOM prevalence over time, rising from 21 to 31% between 1 and 5 years in Kuipers et al. and from 3 to 8% over the same period in Tanabe et al. [13, 22]. Given that BOM may develop gradually after E-POEM, studies with shorter surveillance intervals may fail to identify delayed radiographic changes or late symptom recurrence. Follow-up duration therefore appears to be a key determinant of BOM detection and the true long-term incidence and clinical significance of BOM may be underestimated in the currently available literature [10, 22, 23].

Despite its anatomic distinctiveness, BOM did not demonstrate consistent associations with symptom severity across studies. Most commonly reported symptom associated with BOM included recurrent dysphagia, regurgitation, esophageal stasis, reflux symptoms, and persistent or recurrent achalasia-related symptoms reflected by elevated Eckardt scores. In our study, symptom severity assessed by the Eckardt score was similar between patients with and without BOM at both baseline (approximately 6.7 in both groups) and 3 months follow-up (2.1 in the BOM group and 1.9 in the non-BOM group). In contrast, studies with longer follow-up have reported higher Eckardt scores among patients who developed BOM. Triggs et al. reported mean Eckardt scores of 3 in patients with BOM compared to 2 in those without BOM. In the same cohort, treatment failure defined by an Eckardt score greater than 3 occurred in around 57% of patients with BOM compared with 29% of those without BOM [10]. Similarly, Tanabe et al. stratified patients with BOM by clinical outcome and reported higher mean Eckardt scores in those with clinical failure (3.5) compared with those with clinical success (0.9), with regurgitation occurring more frequently in patients with higher symptom scores [22]. Taken together, these observations support a time-dependent relationship between BOM and symptom severity.

Procedural characteristics, including myotomy length, also did not show clear differences between patients with and without BOM. In our study, mean myotomy length was similar measuring around 10 cm in the BOM group and 11 cm in the non-BOM group, suggesting that length alone may not fully explain BOM development in clinical practice. Using simulations, Halder et al. demonstrated that longer myotomies increase wall deformation and bolus accumulation at the myotomy site, thereby increasing the theoretical risk of BOM. Our findings indicate that clinically performed myotomy lengths overlap between groups. This discrepancy may reflect the multifactorial nature of BOM formation, where myotomy length interacts with esophageal contraction patterns, residual EGJ tone, and wall stiffness rather than acting as an isolated determinant [10, 22, 24].

Mechanistic insights from in-silico modeling showed the potential role of residual EGJ tone, a physiologic correlation of IRP, in the development of BOM. In our study, mean IRP in patients with BOM was approximately 27 mmHg preoperatively and 16 mmHg after E-POEM, indicating residual elevations despite postprocedural reduction [14, 24]. The relationship between residual EGJ outflow resistance and the development of BOM showed mixed findings across studies. Kuipers et al. reported higher postoperative IRP values in patients who subsequently developed BOM compared with those who did not [13]. In contrast, Tanabe et al. did not identify elevated postoperative IRP as an independent risk factor, suggesting that procedural strategies tailored to adequately address spastic segments particularly in type III achalasia may mitigate residual obstruction [22]. Triggs et al., along with commentary by Halland et al., observed that a greater proportion of patients with BOM exhibited IRP values above thresholds associated with residual achalasia, proposing that persistent outflow resistance combined with longer follow-up may contribute to symptom burden and treatment failure over time [10]. These observations raise the possibility that persistent or recurrent elevation in IRP, rather than early postprocedural measurements alone, may be relevant to BOM pathophysiology. Supporting this concept, Krause et al. reported higher post-treatment IRP values in patients with BOM compared with those without, along with a higher prevalence of type III achalasia. In that cohort, although BOM was more frequently observed after LHM than E-POEM, an elevated IRP was accompanied by higher symptom scores [25]. Hence, the need for longitudinal assessment of IRP and esophageal motility to better characterize their relationship with BOM development.

In parallel, our analysis did not demonstrate statistically significant associations between BOM and achalasia subtype. The pooled odds ratios were 0.73 for type I, 0.79 for type II, and 1.38 for type III achalasia. Tanabe et al. did not identify type III achalasia as independent risk factors for BOM, attributing this to the lower prevalence of type III achalasia in Japan and a procedural approach in which myotomy length is adjusted to fully encompass the spastic segment, thereby minimizing residual spasm [22]. Although the point estimate for type III achalasia was numerically higher, this did not reach statistical significance. Prior studies have proposed that type III achalasia predispose patients to BOM. Triggs et al. suggested that limiting myotomy length in type I and II achalasia restricting division to the circular muscle layer or the LES alone may reduce vulnerability to BOM, citing the absence of BOM in patients treated with pneumatic dilation. They further proposed reserving longer, tailored myotomies for type III achalasia to adequately address spastic contractions [10]. Overall, these findings suggest that although subtype-related patterns may exist, the development of BOM is likely multifactorial, with combined influence of procedural factors, esophageal mechanics, and patient-specific characteristics rather than manometric subtype alone [26].

Management of symptomatic BOM remains challenging, and no standardized treatment strategy has been established. While asymptomatic BOM may be managed conservatively, symptomatic cases associated with food retention and recurrent dysphagia despite adequate LES disruption. Traditional approaches have included repeat myotomy or surgical diverticulectomy, though these interventions may not directly address the altered distal esophageal anatomy responsible for impaired bolus clearance [27]. More recently, endoscopic therapies have emerged as minimally invasive alternatives aimed at correcting the anatomic and functional consequences of BOM. These include septal myotomy through a submucosal tunneling approach (B-POEM), which targets residual muscular septa contributing to pouch formation, as well as endoscopic suture exclusion of the saccular dilation to remodel the esophageal lumen [28]. Per-oral direct diverticulotomy (PODD), an ultra–short tunnel technique targeting the central septum of the BOM, has been described as a technically straightforward salvage option with encouraging short-term symptom and physiologic outcomes [29]. Case reports and small series have demonstrated symptom improvement following these strategies, suggesting that restoring luminal geometry and improving esophageal emptying rather than further reducing LES pressure alone may be helpful in successful management. However, current evidence is limited and comparative data regarding durability, patient selection, and optimal techniques are lacking [27, 28].

This study has a few limitations that should be acknowledged. First, the meta-analysis was based on a small number of included studies limiting the ability to determine true incidence rates, generalizability or establish causal relationships. Second, follow-up duration varied across studies, and shorter observation periods may have underestimated the occurrence of BOM, which appears to develop over time. Third, definitions of BOM were not entirely uniform, with reliance on radiographic criteria, endoscopic findings, or both. Two studies defined BOM radiographically as a > 50% increase in distal esophageal diameter on TBE, whereas others required the presence of a distal diverticulum at the prior myotomy site. This variability in diagnostic definitions and imaging interpretation may have contributed to differences in reported prevalence and clinical outcomes across studies. Due to the limited number of available studies and inconsistent reporting methods, subgroup analysis based on BOM definition was not feasible. Fourth, procedural details such as myotomy length, depth, approach, and preservation of sling fibers were not consistently reported, precluding analysis of technical factors that may influence BOM development. Finally, patient-level data were limited, restricting adjustment for potential confounders such as residual motility, postoperative IRP trends, or symptom trajectories. These limitations highlight the need for prospective, longitudinal studies, and detailed procedural reporting to better characterize the natural history and clinical relevance of BOM after E-POEM.

## Conclusions

BOM develops in a minority of patients after E-POEM and appears to be a time-dependent phenomenon. Longer follow-up is required to identify clinically relevant cases and clarify their impact on symptoms and outcomes.
